# Identification of Exosomal miRNAs in Rats With Pulmonary Neutrophilic Inflammation Induced by Zinc Oxide Nanoparticles

**DOI:** 10.3389/fphys.2018.00217

**Published:** 2018-03-13

**Authors:** Yamei Qiao, Xiao Liang, Yingjie Yan, Yake Lu, Di Zhang, Wu Yao, Weidong Wu, Zhen Yan

**Affiliations:** ^1^Department of Occupational and Environmental Health Sciences, College of Public Health, Zhengzhou University, Zhengzhou, China; ^2^Department of Occupational and Environmental Health Sciences, School of Public Health, Xinxiang Medical University, Xinxiang, China; ^3^Department of Occupational and Environmental Health Sciences, School of Public Health, Hainan Medical University, Haikou, China

**Keywords:** zinc oxide nanoparticles, miRNAs, exosomes, pulmonary inflammation, bioinformatics analysis

## Abstract

It has been previously shown that inhaled zinc oxide nanoparticles (ZnO-NPs) can modulate inflammation. MicroRNAs (miRNAs) enclosed in exosomes have been identified as an important signature for inflammatory responses. However, the role of exosomal miRNAs during pathogenic inflammation has not been investigated. Healthy rats were exposed to ZnO-NPs (41.7 nm; 2, 4, and 8 mg/kg) or saline (control) via oropharyngeal aspiration. ZnO-NPs induced significant increases in the serum levels of interleukin 8 (IL-8), interleukin-1 beta (IL-1β), and tumor necrosis factor α (TNF-α), and elevated the number of cells and the percentage of neutrophils in the blood. Moreover, exposure to ZnO-NPs increased the levels of lactate dehydrogenase (LDH) activity and total protein in bronchoalveolar lavage fluid (BALF). Differential profiling of miRNAs in isolated serum exosomes revealed that 16 miRNAs were up-regulated and 7 down-regulated in ZnO-NP-treated rats compared with the controls. Functional and pathway analysis indicated that miRNAs may participate in inflammation directly and indirectly through protein and vesicle-mediated transport or regulation of IL-1, oxidative stress, apoptosis, and autophagy. These results suggest that miRNAs in serum exosomes are involved in pulmonary neutrophilic inflammation induced by ZnO-NPs.

## Introduction

Zinc oxide nanoparticles (ZnO-NPs) are one of the most abundantly produced metal oxide nanoparticles, and have been widely used in industries of cosmetics, paints, textiles, food additives, personal hygiene products, etc., (Fukui et al., 2015). Many *in vitro* and *in vivo* studies (Huang et al., 2015; Morimoto et al., 2016; Chuang et al., 2017) have demonstrated that ZnO-NPs induce airway inflammation in response to pulmonary exposure, and can promote the onset of various respiratory diseases. Moreover, exposure to engineered ZnO-NPs can increase the expression and secretion of neutrophil and pulmonary inflammatory mediators (Larsen et al., 2016; Nemmar et al., 2017). ZnO-NPs have been shown to interfere with zinc homeostasis of the cell, generate excessive reactive oxygen species (ROS), and induce mitochondria dysfunction leading to pulmonary inflammation (Kao et al., 2012; Jeong et al., 2013; Chevallet et al., 2016). One study showed that ZnO-NPs of 20–70 nm in diameter could be internalized by endothelial cells, resulting in a ZnO-induced inflammatory response due to the accumulation of the particles rather than ZnO-released Zn^2+^ (Gojova et al., 2007). Recently, the mechanisms underlying ZnO-NPs toxicity have been shown to depend on the induction of apoptosis and autophagy (Roy et al., 2014; Bai et al., 2017). However, less is known about the underlying regulatory mechanisms of ZnO-NP-induced lung inflammation.

Exosomes are lipid bilayer vesicles of 30–100 nm in size derived from multivesicular bodies after they fuse with the plasma membrane. Exosomes play an intricate role in the initiation and progression of inflammation (Escrevente et al., 2011; Sakha et al., 2016). They were first identified in the early 1980s, but were initially regarded as garbage-bag-wrapped abandoned plasma membranes or membrane molecular fragments (Zhao et al., 2017). It was later found that exosomes are secreted in all biological fluids, including the blood, urine, saliva, cerebrospinal fluid, and *in vitro* cell culture medium (Sakha et al., 2016). Importantly, exosomes are filled with valuable cellular material from parental cells and convey biological signals to surrounding cells when taken up by fusion or by internalization (Di Modica et al., 2017). Exosomes are therefore emerging as important mediators of cell-to-cell communication (Mihelich et al., 2016). Given that intercellular communication is key in inducing and resolving inflammatory responses (Wahlund et al., 2017), exosomes have been associated with the initiation, aggravation, and propagation of inflammation. For example, exosomes isolated from mycobacteria-infected macrophages induces a TLR-dependent inflammatory response (Bhatnagar and Schorey, 2007). Exosomes isolated from *Leishmania donovani* modulate human monocyte cytokine responses to interferon-gamma (IFN-γ) in a bimodal fashion by promoting interleukin 10 (IL-10) production and inhibiting tumor necrosis factor α (TNF-α) (Silverman et al., 2010). Owing to their ability to transport pro-inflammatory molecules and to reach distant organs and compartments, exosomes can trigger an inflammatory response in a context-dependent manner (Chen et al., 2017; Wahlund et al., 2017).

Exosomes play a very crucial role in inflammation due to the nature their cargo molecules, which include protein and genetic material, such as microRNAs (miRNAs) (Sakha et al., 2016). miRNAs are small (18–25 nucleotides), single-stranded, and highly conserved non-coding RNAs, and are able to suppress the translation and/or initiate the degradation of target mRNAs (Jung et al., 2016; Mihelich et al., 2016), thus reducing protein expression. miRNAs are differentially enriched in exosomes in a cell-type-dependent fashion, and can be carried by donor cells, released into the extracellular environment, then transferred into recipient cells to regulate the gene expression of distant cells (Squadrito et al., 2014; Zhao et al., 2015). Exosomes freely circulate in the blood, which contains billions of exosomes per microliter (Zhou et al., 2017), and are regarded as the predominant form of circulating miRNAs. Furthermore, miRNAs are protected by encapsulation with exosomes, making the oligonucleotides extremely stable and readily extracted from various types of cell lines or tissues (How et al., 2015; Sun et al., 2017). Moreover, recent studies have shown that miRNAs play a central role in multiple aspects of lung inflammation and disease pathogenesis (Alipoor et al., 2016). Therefore, the stability, cell type specificity, and high bioavailability make exosomal miRNAs valuable factors in elucidating the mechanisms of toxicity and disease progression, and discovering novel therapeutic treatments.

The biological functions of serum exosomes in normal or pathological conditions remain unclear. In the present study, we investigated the onset of pulmonary inflammation induced by ZnO-NPs. miRNA profiles in serum exosomes were analyzed via microarray to identify signature miRNAs involved in airway inflammation. Furthermore, we analyzed the function and regulation of putative target genes using a bioinformatic approach to further characterize signaling pathways associated with pulmonary inflammation induced by ZnO-NPs.

## Materials and methods

### ZnO-NP suspension

Uncoated ZnO-NPs were purchased from Nanjing High Technology of Nano Co. (Nanjing, China), and were previously characterized. The purity of ZnO-NPs was 98.8%, with an average size of 41.7 nm and a surface area of 23.6 m^2^/g (Yan et al., 2014). Stock suspensions of ZnO-NPs (1 mg/mL) were prepared in phosphate-buffered saline (PBS, pH7.4) and sonicated for 120 s prior to each oropharyngeal aspiration.

### Oropharyngeal aspiration of ZnO-NPs suspension

Specific-pathogen-free (SPF) male Wistar rats (body weight 180 ±15 g) were obtained from the Experimental Animal Center of Henan province. Rats were raised in a certified laboratory animal facility with a barrier system, housed in a light- and temperature-controlled room, and fed *ad libitum* with chow and distilled water. After 1 week of acclimation, 24 rats were randomized into 4 groups (6 rats per group). Three groups were oropharyngeally aspirated with varying doses (2, 4, and 8 mg/kg) of ZnO-NPs suspension once a day for a total of 3 days. Rats in the control group were aspirated with saline. Twenty-four hours after the last aspiration, rats were anesthetized using 5% chloral hydrate (10 mL/kg). Whole blood was drawn from the abdominal aorta, of which one part was used for determination of blood cell count and the other part stored in a refrigerator at 4°C for 1 h, followed by centrifugation at 1,000 × g for 10 min. The serum was separated, aliquoted, and stored at −80°C until analysis. The trachea was cannulated with the left bronchus clamped. Lung lavage was conducted with the right lungs 3 times with 3 mL of sterile saline, and the recovered fluids were pooled together. Similar amounts of bronchoalveolar lavage fluids (BALFs) were obtained from each group. BALFs were centrifuged (1,000 × g for 10 min at 4°C) and the supernatant was stored at −80°C for further analysis. The left lung was fixed with paraformaldehyde at room temperature for pathological examination. All experiments were performed following guidelines for care and use of animals from Zhengzhou University and approved by the Animal Experimentation Ethics Committee of Zhengzhou University.

### Measurement of serum levels of IL-8, IL-1β, and TNF-α

Serum levels of interleukin-8 (IL-8), IL-1β, and tumor necrosis factor α (TNF-α) were measured with enzyme-linked immunosorbent assay (ELISA) kits (Shanghai Blue Gene Biotech CO., Shanghai, China) per manufacturer's protocol, and the optical density (OD) was detected at 450 nm using a microplate reader (Tecan Sunrise; Germany).

### Measurement of lactate dehydrogenase (LDH) protein levels and activity in BALF

LDH activity was measured with an LDH assay kit (Beyotime, China) using a wavelength of 450 nm per manufacturer's instructions. LDH protein levels in BALF were estimated using a bicinchoninic acid assay kit (Beyotime, China).

### Histopathological examination

Unlavaged left lungs were fixed in 10% paraformaldehyde at room temperature overnight. After fixation, paraffin-embedded lung tissues were cut into sections of 5 μm and stained with hematoxylin and eosin (H&E). The stained sections were examined by a histopathologist for inflammation and morphological changes using light microscopy in a double blinded fashion.

### Extraction of exosomes from serum

Rats treated with 4 mg/kg ZnO-NPs or saline (3 animals per group) were chosen for exosome study. ExoRNeasySerum/Plasma Kit (Qiagen, Hilden, Germany) was used for exosome extraction per manufacturer's protocol. Using this kit, we concentrated the extracellular RNA into a final volume of 14 μL diluted with water.

### Morphological examination of exosomes with transmission electron microscopy (TEM)

The morphology of exosomes was assessed by TEM. Approximately 10 μL of exosomes were loaded into carbon-coated 220 mesh copper grids and allowed to adhere for 2 min. The adsorbed exosomes were then negatively stained with 1% phosphotungstic acid and dried at room temperature for 60 min. Subsequently, the exosomes were observed under TEM (JEM-1400; JEOL Ltd., Tokyo, Japan) at an acceleration voltage of 80 kV.

### Western blot

Exosomal proteins were separated via sodium dodecyl sulfate-polyacrylamide gel electrophoresis (SDS-PAGE) and transferred to PVDF membranes (Millipore Corp. Bedford, MA). The membranes were blocked in tris-buffered saline (TBS) containing 5% nonfat milk for 2 h, then incubated with specific primary antibodies to CD63 (Abgent, San Diego CA) and Alix (Cell Signaling Technology, Danvers, MA) at 4°C overnight. The membranes were washed 3 times with TBS containing 0.1% Tween-20 (TBST) and re-probed with HRP-conjugated secondary antibodies for 1.5 h at room temperature. The membranes were washed 3 times with TBST, and the bands were visualized by enhanced chemiluminescence (ECL).

### miRNA microarray assay and bioinformatics analysis of target genes

miRNA profiling of serum exosomes from rats treated with ZnO-NPs or saline was performed by the Professional Oebiotech Corporation (Shanghai, China, http://www.oebiotech.com). Briefly, the extracted RNA was labeled and hybridized to an Agilent-070154 Rat miRNA Microarray V21.0 8 × 15K (Agilent). Genespring software (version 13.1, Agilent Technologies) was employed to normalize the raw data, and differentially expressed miRNAs (DEmiRNAs) were identified. The threshold for up- or down-regulated genes was a fold change of ≥1.5 and a *P* ≤ 0.05. Target genes of DEmiRNAs were selected through examination of the overlapped intersection from two databases (Targetscan and microRNAorg). The putative genes were subjected to functional and pathway enrichment analysis using Gene Ontology (GO) and the Kyoto Encyclopedia of Genes and Genomes (KEGG). The threshold of significance was defined as *P* ≤ 0.05 for both GO and KEGG analyses. The potential regulatory relationships between miRNAs and target genes were analyzed using Cytoscape software (http://www.cytoscape.org/).

### Statistical analysis

All statistical analyses were performed with SPSS 21.0 statistical software. Data are presented as means ± SD. Comparisons between groups were performed by one-way analysis of variance (ANOVA), followed by *post-hoc* analysis using the least significant difference (LSD). *P* < 0.05 was considered statistically significant.

## Results

### ZnO-NPs modulate the levels of inflammatory cytokines and increase the number of white blood cells

**Figure 1:**
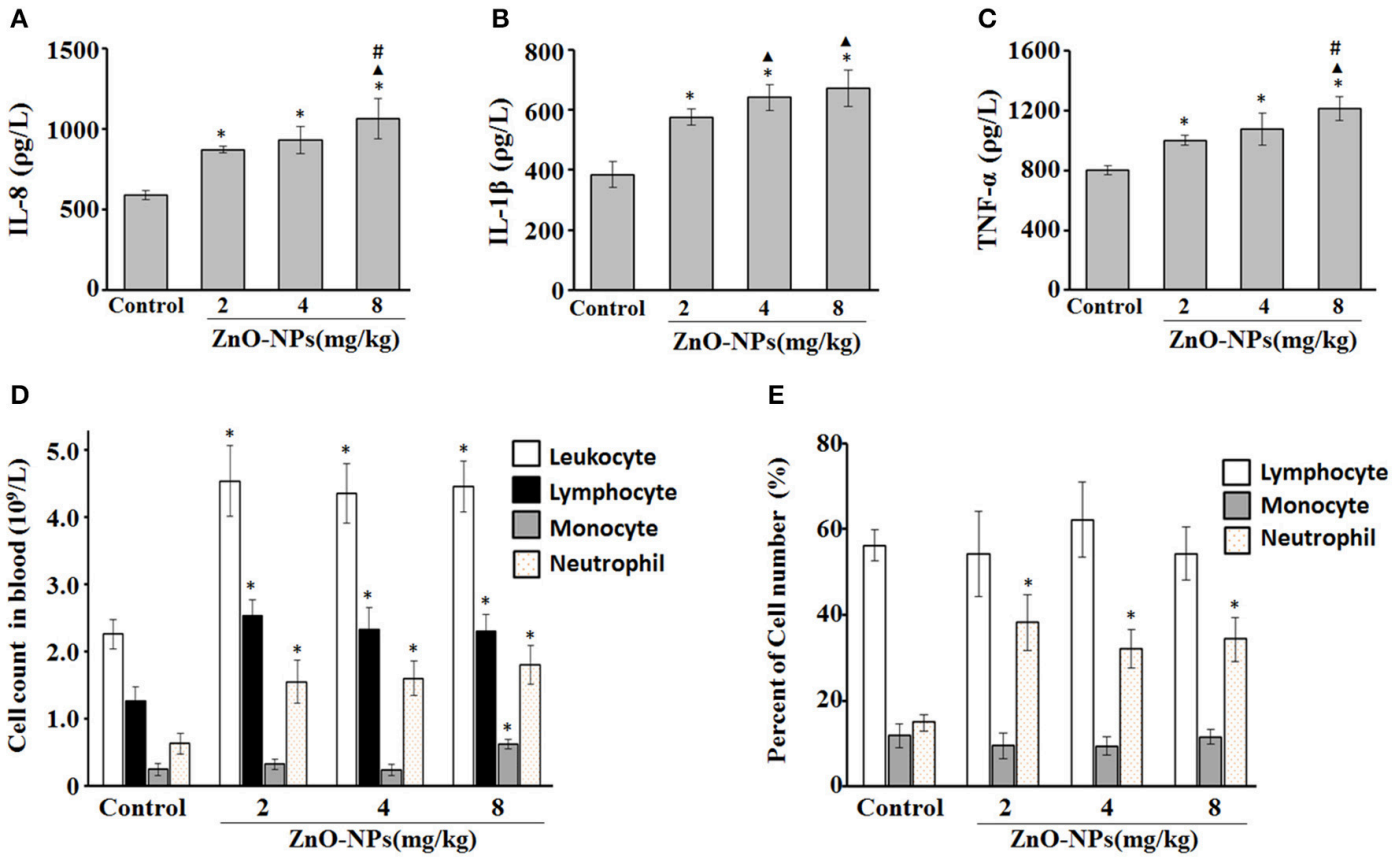
Characterization of inflammatory cytokines and cell number after aspiration with ZnO-NPs in rats. Serum levels of IL-8 **(A)**, IL-1β **(B)**, and TNF-α **(C)**. **(D)** Cell counts (leukocyte, lymphocyte, monocyte, and neutrophil) and **(E)** the percentage of cells in blood. ^*^Compared to control group, *P* < 0.05; ▴ compared to 2 mg/kg group, *P* < 0.05; ^#^compared to 4 mg/kg group, *P* < 0.05.

Compared with the control group, exposure to ZnO-NPs at 2, 4, and 8 mg/kg induced significant increases in IL-8 production (*P* < 0.05; Figure 1). However, the levels of IL-1β (Figure 1B) and TNF-α (Figure 1C) were also significantly elevated in response to ZnO-NP exposure. Furthermore, ZnO-NP exposure increased the cell counts of blood leukocytes, lymphocytes, monocytes, and neutrophils compared with the control, while the percentage of neutrophils was also significantly elevated (Figures 1D,E).

### ZnO-NPs induce airway inflammation

**Figure 2:**
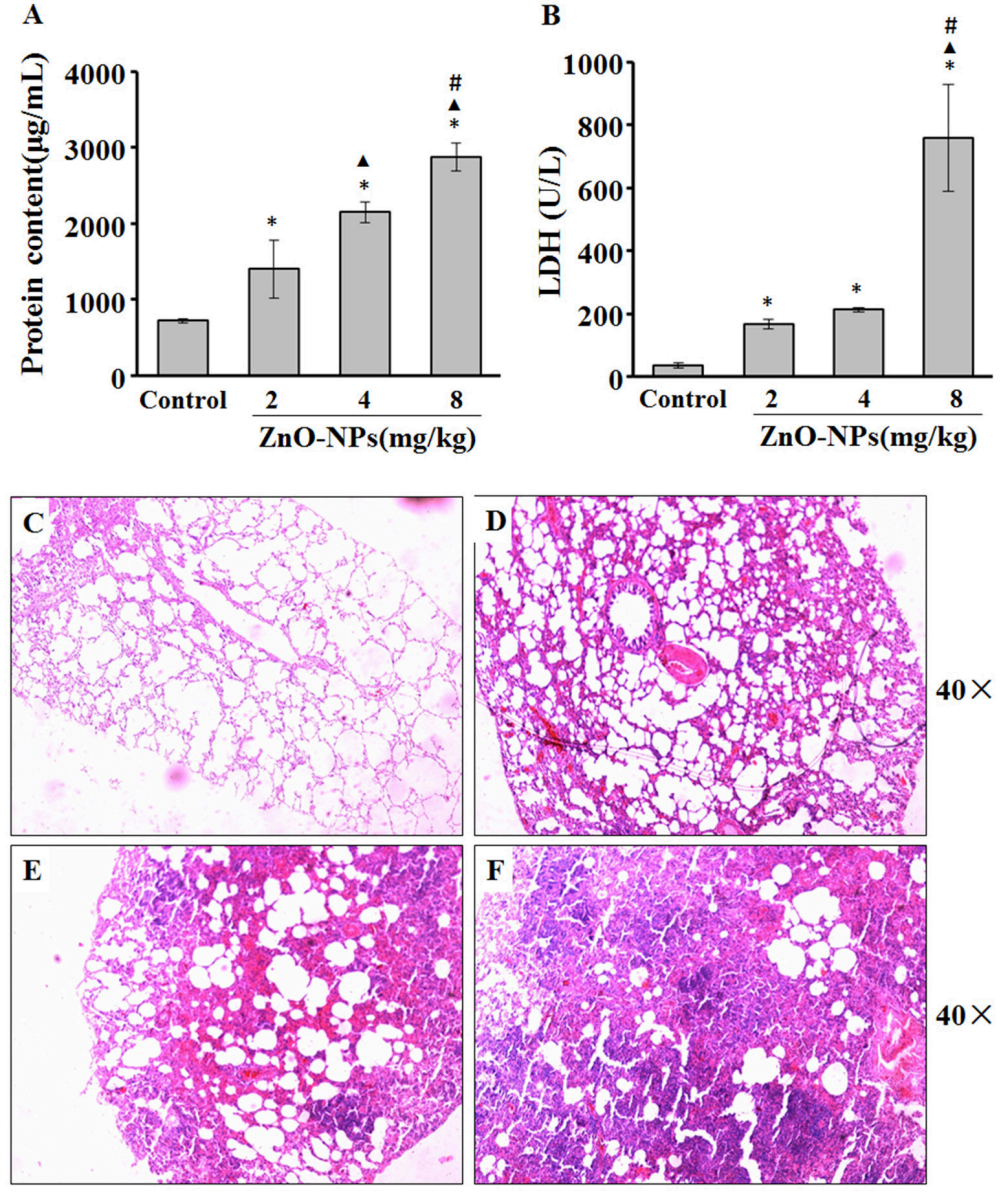
ZnO-NP-induced pulmonary inflammation. After last administration of either ZnO-NP or saline (control), the rats were anesthetized and BALF was collected to measure total proteins **(A)** and LDH **(B)** activity. Lungs from rats exposed to saline **(C)** or ZnO-NP **(D–F)** were sectioned and stained with hematoxylin and eosin (H&E; *n* = 6). ^*^Compared to control group, *P* < 0.05; ▴ compared to 2 mg/kg group, *P* < 0.05; ^#^compared to 4 mg/kg group, *P* < 0.05.

We observed a dose-dependent increase in the protein levels of BALF from rats aspirated with ZnO-NPs (Figure 2A). Additionally, LDH activity was significantly elevated in the BALF of rats exposed to ZnO-NPs compared with the control (Figure 2B).

There was minimal change of the alveolar structure in rats aspirated with saline. In contrast, mild inflammatory cell infiltration and exudate and edema formation were observed in lungs exposed to 2 mg/kg ZnO-NPs (Figure 2D). We also observed solid changes, severe infiltration, congestion, and exudate of inflammatory cells in rats exposed to 4 mg/kg ZnO-NPs (Figure 2E). Rats exposed to 8 mg/kg ZnO-NPs showed severe congestion and edema, and complete consolidation of the lung (Figure 2F). Based on these results, we administered 4 mg/kg of ZnO-NPs for all other experiments.

### ZnO-NPs alter the morphology of exosomes

**Figure 3:**
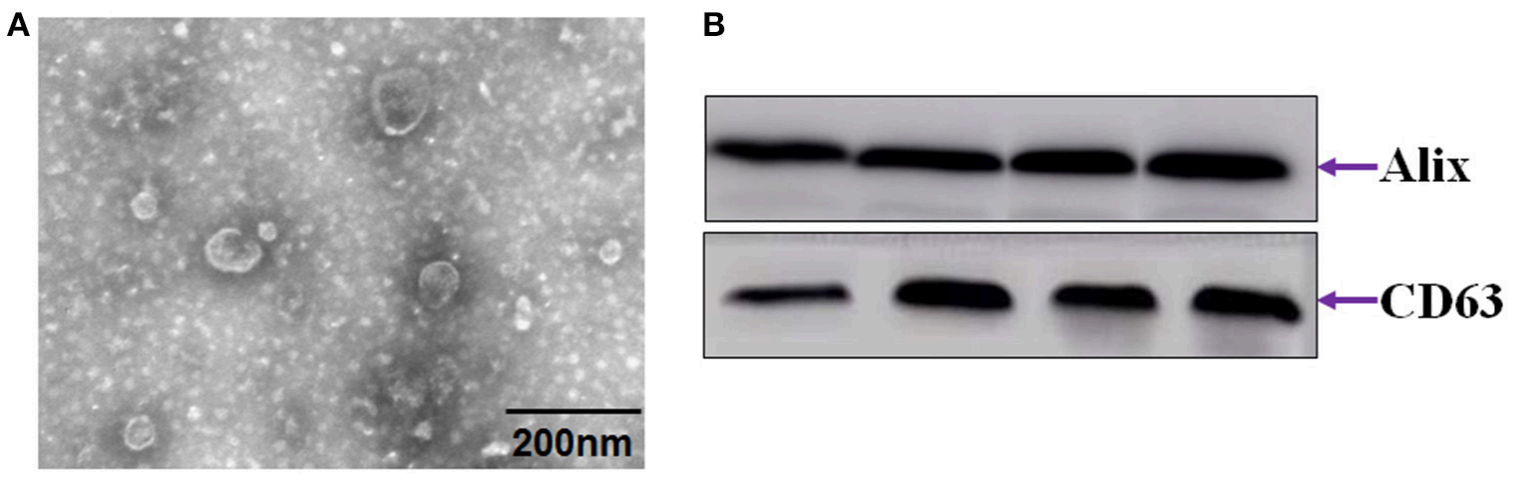
Identification of exosomes from serum. **(A)** Electron microscope images of exosomes. **(B)** Immunoblot of CD63 and Alix.

Most exosomes contain diverse proteins such as heat shock proteins, certain members of the tetraspanin superfamily of proteins, especially CD9, and CD63, and endosomal sorting required for transport proteins, TSG-101 and ALIX, which enable the identification of exosomes (Khushman et al., 2017; Menay et al., 2017). Figure 3A shows exosomes presented as round- (“cup”) or oval-shaped (“dish”) with a diameter of 30 to 100 nm. To confirm the presence of exosomes, we assessed the expression of 2 common exosome markers, CD63 and Alix (Figure 3B).

### ZnO-NPs modulates the expression of a subset of miRNAs

**Figure 4:**
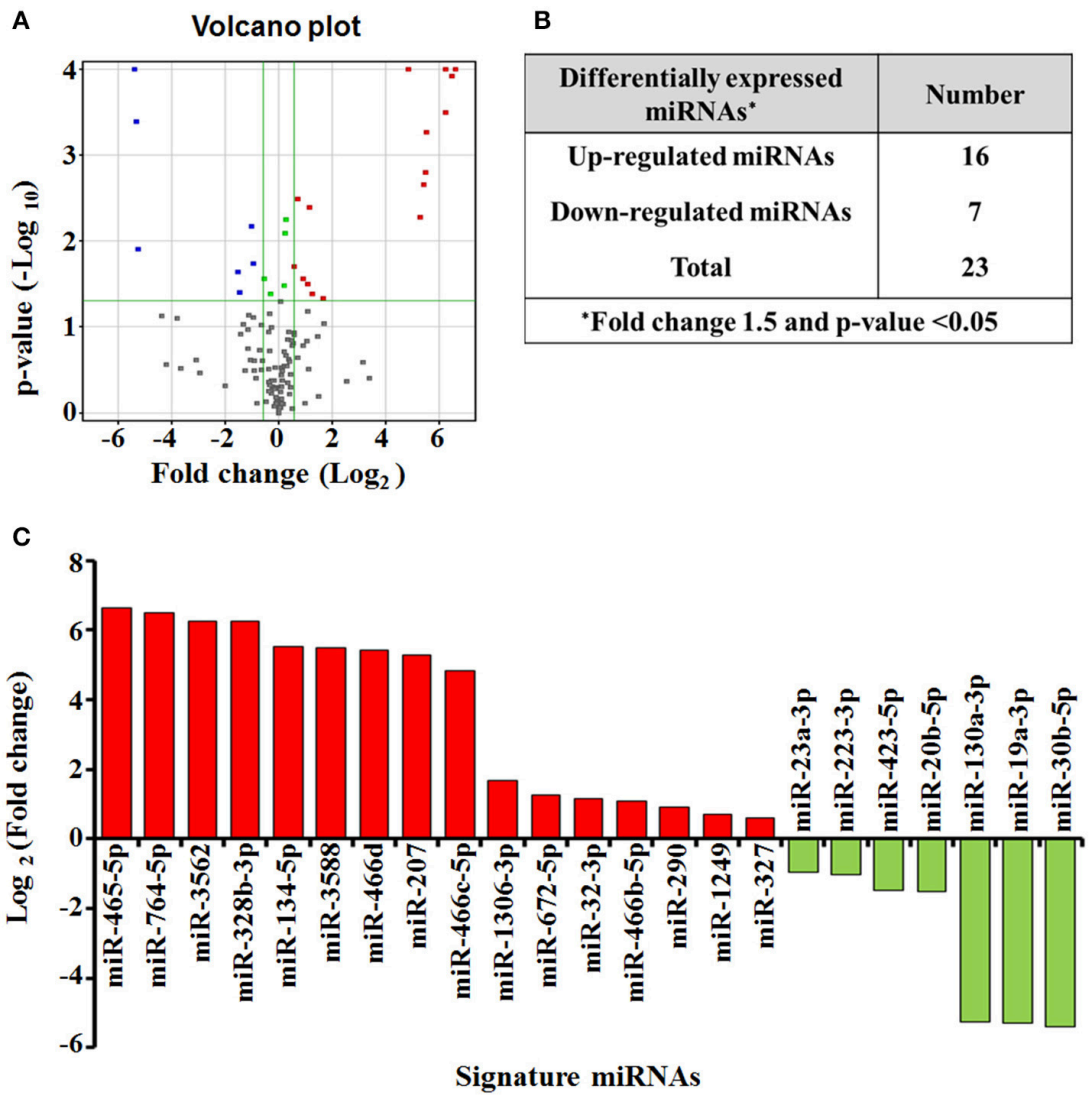
miRNA expression profiling in response to airway inflammation induced by ZnO-NPs. Differentially expressed miRNAs (*P* < 0.05) were analyzed by hierarchical clustering of log 2 value. In the volcano plot **(A)** for differential gene expression, red and blue colors indicate significantly up- or down-regulated miRNAs, respectively. Green dots represent no significant change. The number **(B)** and profile **(C)** of the differentially expressed miRNAs are shown.

To identify candidate exosomal miRNAs associated with pulmonary inflammation induced by ZnO-NPs, we used the Agilent Rat miRNA Microarray for expression profiling of miRNAs in serum exosomes. A total of 23 differentially expressed miRNAs (DEmiRNAs) in rats exposed to ZnO-NPs were initially screened (Figure 4A), including 16 up-regulated and 7 down-regulated miRNAs (Figure 4B). Among the DEmiRNAs identified, 12 of them exhibited more than a 5-fold alteration in serum exosomes from rats treated with ZnO-NPs. Detailed information of the 23 DEmiRNAs is summarized in Figure 4C.

### Target gene prediction and pathway enrichment analysis of DEmiRNAs

**Figure 5:**
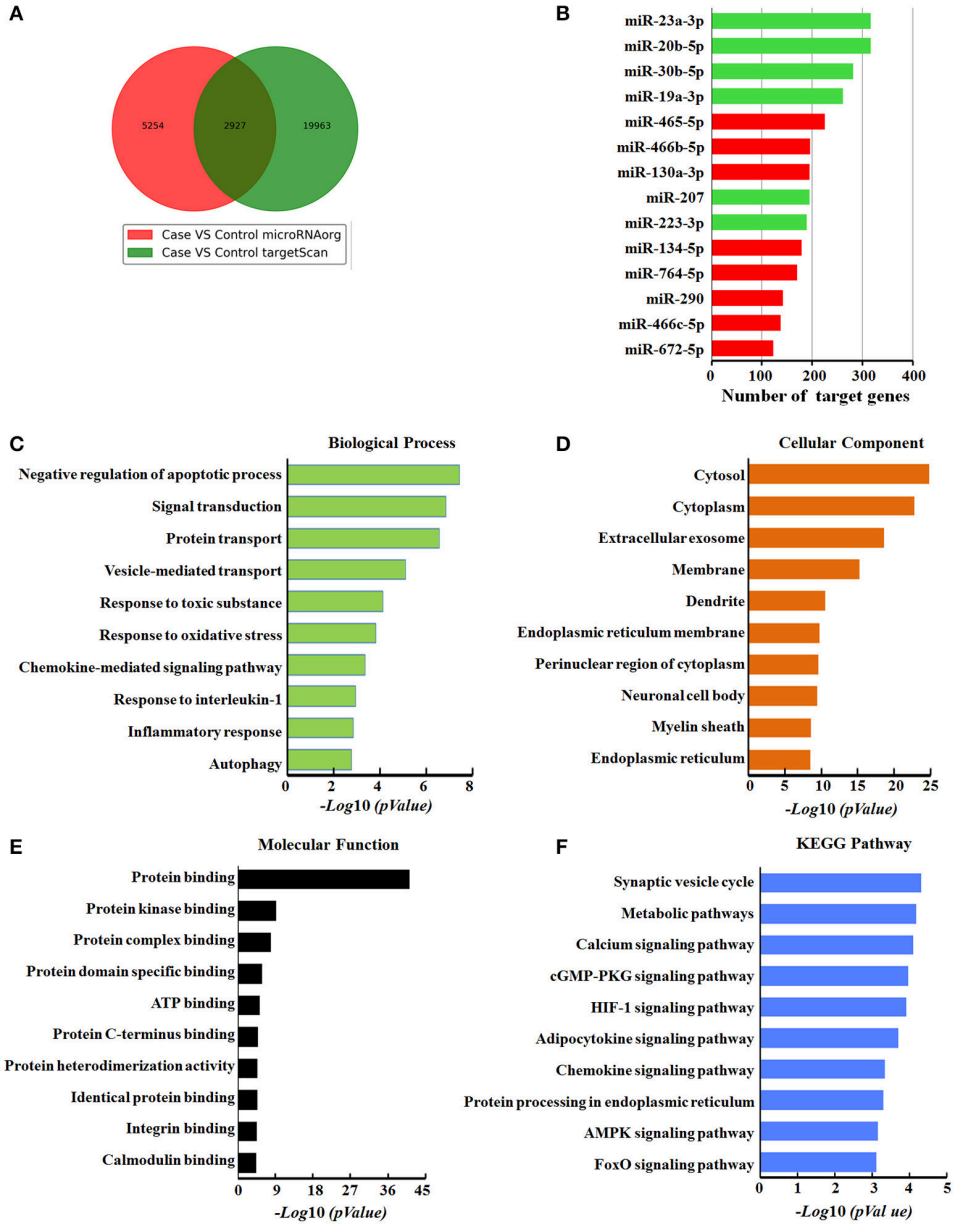
Bioinformatics analysis of target genes modulated by differentially expressed miRNAs in response to ZnO-NPs treatment in rats. **(A)** Venn diagram showing the overlap of candidate target genes. **(B)** Bar charts showing the number of target genes for up- or down-regulated miRNAs. Red and green bars indicate the number of target genes for up- and down- regulated miRNAs, respectively. **(C)** Biological process, **(D)** cellular components, and **(E)** molecular function assessed by Gene Ontology (GO) analysis. The chart fragments represent the number of target genes associated with each term. **(F)** KEGG pathway enrichment analyses for target genes. The terms are listed on the *Y*-axis, while the *P*-values are listed on the *X*-axis.

To further understand the role of DEmiRNAs during airway inflammation, we searched for their putative target genes using miRNA prediction databases Targetscan and microRNA.org. We found that 14 of the DEmiRNAs identified in our screen can regulate 2927 common putative target genes (Figure 5A), while 9 miRNAs, including miR-1249, miR-1306-3p, miR-32-3p, miR-327, miR-328b-3p, miR-3562, miR-3588, miR-423-5p, and miR-466d, had no common predicted genes. We assumed that 8 up-regulated miRNAs might target 1367 genes, while the 6 down-regulated miRNAs may target 1560 genes. Among the DEmiRNAs, down-regulated miRNAs, including miR-23a-3p and miR-20b-5p, had the most target genes with 317 and 316, respectively (Figure 5B).

Next, to identify the functional role of these DEmiRNAs, we performed enrichment analysis for their putative target genes using GO terms. The GO terms associated with these target genes were related to the response to toxic substances, cellular response to apoptosis, vesicle-mediated transport, oxidative stress, interleukin-1 (IL-1), and autophagy, indicating that these DEmiRNAs are likely related to pulmonary inflammation. The target genes mediated by these DEmiRNAs are also primarily located in extracellular exosomes, cellular membranes, and cytoplasm, (Figure 5D), with protein binding as the most dysfunctional molecular function annotation (Figure 5E).

To further examine the biological function of the DEmiRNAs, we investigated the KEGG pathways of their target genes (Figure 5F). The most enriched dysfunctional pathway included synaptic vesicle formation. HIF-1 and chemokine signaling pathways were also included in the enriched subsystems. Our pathway analysis partly reflects the function of the signature miRNAs, and signal-related function was highlighted among all the subsystems, which was consistent with GO function analysis of the target genes.

### Construction of a regulatory network for integrated miRNA-target genes

**Figure 6:**
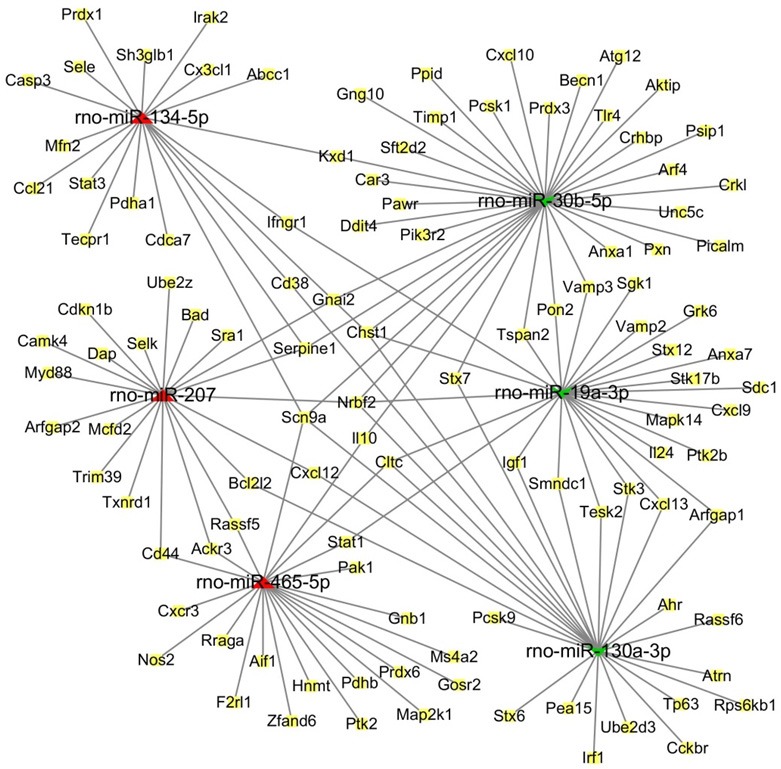
The regulatory network of miRNA-target genes in response to ZnO-NP exposure. The predicted interaction network of miRNAs and target genes was constructed by software Cytoscape. Red triangles refer to up-regulated miRNAs, while green concave quadrilaterals represent down-regulated miRNAs. Yellow squares indicate the target genes.

Based on functional and pathway analysis and related literature examination, we selected miR-134-5p, miR-207, and miR-465-5p to represent the up-regulated miRNAs, and miR-30b-5p, miR-19a-3p, and miR-130a-3p to represent the down-regulated miRNAs. We investigated the associations between the DEmiRNAs and target genes by construction a regulatory network for miRNA-target genes using Cytoscape software (Figure 6). The core of the interactive network of target genes included *Chst1, Nrbf2*, and *Scn9a*. In our network, *Chst1* (carbohydrate sulfotransferase 1) is regulated by four miRNAs, including miR-30b-5p, miR-19a-3p, miR-130a-3p, and miR-134-5p, while *Nrbf2* (nuclear receptor binding factor 2) is regulated by miR-30b-5p, miR-19a-3p, miR-130a-3p, and miR-207. Finally, *Scn9a* is modulated by miR-30b-5p, miR-130a-3p, miR-134-5p, and miR-465-5p.

## Discussion

In this study, pulmonary inflammation in rats induced by oropharyngeal aspiration of ZnO-NPs was represented by increases in the levels of TNF-α, neutrophils, LDH activity and protein levels in BALF, and inflammatory pathological characteristics. On this basis, serum exosomes from ZnO-NP-exposed rats were extracted, and miRNAs were identified and their expression levels were assessed using miRNA microarrays. 23 DEmiRNAs were identified by sequencing and analyzed by bioinformatics. The gene prediction and annotation indicated that these DEmiRNAs participate in the onset of inflammation through different biological pathways. To our knowledge, this is the first study of its kind to explore and analyze the function of exosomal miRNAs in inflammation induced by ZnO-NPs.

Metal fume fever is an acute occupational disease caused by the inhalation of metal fumes, particularly zinc oxide in nano-scale sized fractions (Chuang et al., 2014). Inhalation is the main pathway of ZnO exposure in occupational environments. The majority of *in vivo* studies on ZnO-NP toxicity are predominantly based on exposure to particle suspensions via intratracheal instillation or pulmonary aspiration (Chuang et al., 2014). Ho et al. reported that inhalation of ZnO-NPs (35 nm) induced acute inflammation in Sprague-Dawley rats, and showed that both the mass and surface area of the particles affected the cell number and percentage of neutrophils in the BALF (Ho et al., 2011). Exposure to occupationally relevant ZnO-NPs via intratracheal instillation in Sprague-Dawley rats increased the number of total cells, including neutrophils, and the levels and activity of LDH in BALF, implying that exposure to ZnO-NPs causes neutrophilic inflammation in the lungs (Chuang et al., 2014). In the present study, we showed that aspirated ZnO-NPs similarly caused an increase in neutrophils accompanied with elevated levels of IL-8, IL-1β, and TNF-α in the blood. An increase in the number of circulating neutrophils is a sign of an acute systemic inflammatory response to pulmonary injury caused by ZnO (Jacobsen et al., 2015). This observation was further supported by the histological findings in ZnO-NP-induced neutrophilic inflammation.

Recent studies have demonstrated that exosomal miRNAs play a critical role in regulating inflammation. Exosomes can enrich, encapsulate, and stabilize miRNAs in serum. Serum lacking exosomes still contains a certain amount of miRNA, but the content is significantly lower than that which contains exosomes. Upon the release of miRNA-containing exosomes into the extracellular environment and uptake by recipient cells, miRNAs can modulate various biological processes by regulating the expression of multiple target genes (Sun et al., 2017). One study found that miRNAs involved in chicken ovarian granulosa cell development are regulated by ZnO-NPs (Zhao et al., 2016). Our study found a total of 23 DEmiRNAs in exosomes from ZnO-NP-treated rats, including 16 up- and 7 down-regulated miRNAs. To gain insight into the biological functions of these DEmiRNAs, we predicted the miRNA target genes using miRNA-target-predicting software (microRNAorg and TargetScan). To reduce false positives, target genes located in both databases were chosen for further analyses. After subsequent analysis, 8 up-regulated miRNAs were associated with 1367 putative target genes, while 6 down-regulated miRNAs were linked to 1560 genes. To further understand the role of DEmiRNAs, enrichment analysis was performed on all 2927 predicted target genes using GO terms and KEGG analysis. Our results showed that these target genes are involved processes like the response to apoptosis, vesicle-mediated transport, oxidative stress, chemokine-mediated signaling, IL-1 signaling, inflammation, and autophagy. IL-1β is a strong pro-inflammatory mediator and is crucial for pulmonary inflammation induced by nanoparticles (Tsugita et al., 2017). NLRP3 inflammasome activation is an important signal leading to activation of caspase-1, which subsequently processes pro-IL-1β into mature IL-1β. We previously found ZnO-NPs induce IL-1β expression via the NLRP3 inflammasome, which is activated by ROS, in A549 cells (Liang et al., 2017). Therefore, we infer that these DEmiRNAs might affect the onset of pulmonary inflammation by regulating the cellular response to oxidative stress and IL-1β signaling.

To further characterize the role of exosome-specific DEmiRNAs, we focused on those whose expression was modulated the most by ZnO-NPs. Among these DEmiRNAs, 6 up- or down-regulated miRNAs were chosen for further analysis, including miR-134-5p, miR-207, miR-465-5p, miR-30b-5p, miR-19a-3p, and miR-130a-3p. miR-207 has been previously shown to enhance radiation-induced apoptosis by directly targeting Akt3, while targeting miR-207 protected cochlea hair cells from ionizing radiation (Tan et al., 2014). Plasma miR-134 levels are significantly higher in the patients with acute pulmonary embolism (APE) compared with healthy subjects or patients without APE (Xiao et al., 2011). Additionally, miR-134 was found to be significantly down-regulated in A549/cisplatin multidrug resistance lung adenocarcinoma cells, and its expression levels modulate the proliferation, apoptosis, and invasion of lung adenocarcinoma cells (Li et al., 2017). Overexpression of miR-130a regulates C/EBP-ε protein expression levels in both murine and human granulocytic precursors. Introduction of a C/EBP-ε mRNA lacking a binding site for miR-130a restored both C/EBP-ε production, expression of Camp and Lcn2, and resulted in granulocytic precursors with a more mature phenotype, indicating that miR-130a is important for the regulation of C/EBP-ε expression during granulopoiesis (Larsen et al., 2014). Furthermore, miR-130a down-regulation has been shown to increase the expression of HDAC3 in peripheral blood mononuclear cells (PBMCs), and loss-of-function of miRNA-130a promotes PBMCs apoptosis. In addition, miR-130a overexpression decreases, whereas miR-130a inhibition increases, the expression levels of TNF-α in PBMCs (Ma et al., 2015). It is clear that inflammatory signaling can be controlled by these exosome-specific miRNAs (Alipoor et al., 2016). Interestingly, MathéE et al. found that inflammatory signaling can regulate miRNA expression levels in cooperation with nitric oxide and p53 (Mathe et al., 2012). However, the exact mechanisms linking inflammation and miRNA expression are still unclear.

Our miRNA-target gene regulatory network suggests that a single miRNA can target multiple genes, and that different miRNAs regulate the same gene. The core of this interaction network includes *Chst1, Nrbf2*, and *Scn9a*. *Chst1* is modulated by four miRNAs, including miR-30b-5p, miR-19a-3p, miR-130a-3p, and miR-134-5p. *Chst1* encodes the enzyme CHST1 (carbohydrate sulfotransferase 1), a golgi-localized, transmembrane sulfotransferase that sulfonates specific O-linked carbohydrate side chains on lipids and proteins (Lynch et al., 2014). CHST1 may be expressed by neutrophils and is associated with the generation of L-selectin ligands. To this point, leukocytes have been shown to express L-selectin ligands that are critical for leukocyte-leukocyte interactions (Tu et al., 1999). CHST1 was proposed to contribute to the generation of optimal L-selectin ligands in vascular endothelial cells during inflammation (Li et al., 2001). Additionally, in our study, *Nrbf2* was regulated by miR-30b-5p, miR-19a-3p, miR-130a-3p, and miR-207. Nrbf2 (nuclear receptor binding factor 2) is orthologous to Atg38 in mammals, and has been identified as a subunit of the macroautophagic/autophagic class III phosphatidylinositol 3-kinase complex I (PI3KC3-C1) (Ma et al., 2017), which is central to autophagy initiation (Young et al., 2016). Autophagy, an intracellular degradation system that is associated with the maintenance of cellular homeostasis, plays a key role in inflammasome inactivation (Chen et al., 2016) and inflammatory diseases. Autophagy decreases pro-inflammatory signals by eliminating intracellular organisms, degrading pro-inflammatory signals, and controlling cytokine production and release (Messer, 2017). Nrbf2 has been reported to be critical for the induction of autophagy, as autophagosome formation is blocked in Nrbf2-knockdown cells (Cao et al., 2014). In contrast, some studies have indicated that Nrbf2 could suppress autophagy (Zhong et al., 2014). However, the function of these miRNAs and their target genes in ZnO-NP-induced inflammation remain unclear. Further understanding of these regulatory mechanisms will improve the diagnosis and prevention of pulmonary inflammation mediated by ZnO-NPs.

## Conclusions

Pulmonary exposure to ZnO-NPs induced airway neutrophilic inflammation in rats. During this process, 23 DEmiRNAs in serum exosomes were identified, of which 16 were up-regulated and 7 were down-regulated. The DEmiRNAs (e.g., miR-134-5p, miR-207, miR-465-5p, miR-30b-5p, miR-19a-3p, and miR-130a-3p) and common target genes, such as Chst1 and Nrbf2, may be strongly associated with the pulmonary inflammation induced by ZnO-NPs. These data lay the foundation for the further analysis of the interaction among signature miRNAs and target genes during ZnO-NPs-mediated toxicity. This study also provides novel evidence that exposure to ZnO-NPs induces pulmonary inflammation through modulation of miRNA expression. However, because of our limited knowledge of miRNAs in serum exosomes, more studies are needed to confirm these findings.

## Author contributions

YQ, XL, and YY carried out main part of the studies. YL and DZ collected and analyzed the data. ZY designed the project and drafted the manuscript. WY participated in the design of the study. WW revised the manuscript. All authors reviewed the manuscript.

### Conflict of interest statement

The authors declare that the research was conducted in the absence of any commercial or financial relationships that could be construed as a potential conflict of interest. The reviewer JSK and handling Editor declared their shared affiliation.
